# *Acanthosaura aurantiacrista* (Squamata: Agamidae), a new long horn lizard from northern Thailand

**DOI:** 10.3897/BDJ.8.e48587

**Published:** 2020-05-15

**Authors:** Poramad Trivalairat, Kirati Kunya, Lawan Chanhome, Montri Sumontha, Taksa Vasaruchapong, Nirut Chomngam, Krittiya Chiangkul

**Affiliations:** 1 Animal Systematics and Ecology Speciality Research Unit, Department of Zoology, Faculty of Science, Kasetsart University, Bangkok, Thailand Animal Systematics and Ecology Speciality Research Unit, Department of Zoology, Faculty of Science, Kasetsart University Bangkok Thailand; 2 Nakhonratchasima Zoo, 111 M.1, Ratchasima-Pak Tongchai Rd., Chaimongkol, Muang Nakhonrajsima, Thailand Nakhonratchasima Zoo, 111 M.1, Ratchasima-Pak Tongchai Rd., Chaimongkol Muang Nakhonrajsima Thailand; 3 Head of Snake Farm Queen Saovabha Memorial Institute The Thai Red Cross Society 1871 Rama IV Rd., Bangkok, Thailand Head of Snake Farm Queen Saovabha Memorial Institute The Thai Red Cross Society 1871 Rama IV Rd. Bangkok Thailand; 4 Ranong Marine Fisheries Station, 157 M. 1 Saphan Pla Road, Pak Nam, Muang, Ranong, Thailand Ranong Marine Fisheries Station, 157 M. 1 Saphan Pla Road, Pak Nam, Muang Ranong Thailand; 5 54 M. 2, Tha Pha Subdistrict, Ban Pong District, Ratchaburi, Thailand 54 M. 2, Tha Pha Subdistrict, Ban Pong District Ratchaburi Thailand

**Keywords:** rainforest, Thanon Thong Chai Mountain Range, northern region, ND2

## Abstract

**Background:**

In Thailand, five species of *Acanthosaura* have been recorded so far, including *Acanthosaura armata* from the southern region, *A. cardamomensis* from the eastern region, *A. crucigera* from the western region, *A. lepidogaster* from the northern region and *A. phuketensis* from the Phuket Island and south-western region. However, comprehensive studies of diversity patterns and distribution of *Acanthosaura* are still lacking in some areas and need further information for designating areas of special conservation importance and nature protection planning in Thailand.

**New information:**

*Acanthosaura aurantiacrista* is a new species of long-horned lizard of the genus *Acanthosaura* from northern Thailand. It is distinguished from all other species of *Acanthosaura* by a dagger-like nuchal spine with yellowish-orange colouration in females, bright yellow colouration in males and a combination of other morphological characters: a greater tail length to snout-vent length ratio; a larger postorbital spine, nuchal spine, dorsal spine and occipital spine compared to its head length; a smaller diastema to snout-vent length ratio; a greater number of subdigital lamellae on the fourth finger and fourth toe; and a larger gular pouch than other *Acanthosaura* species. Analysis of mitochondrial ND2 gene sequences revealed a sister clade between the *A. aurantiacrista* lineage and the *A. crucigera* lineage with a 100% probability of divergence, according to Bayesian analysis and strong support value for Maximum Likelihood analysis. The pairwise distance ranged from 13.8-15.0% between *A. aurantiacrista* and *A. cardamomensis*, 10.9-14.5% between *A. aurantiacrista* and *A. crucigera* and 0-1.2% amongst *A. aurantiacrista* populations. The discovery of this lizard increases the known endemic herpetological diversity and underscores the importance of conservation in the mountain rainforest region of northern Thailand.

## Introduction

Currently, fourteen species of agamid lizards of the genus *Acanthosaura* Gray, 1831 are recognised, ranging from Myanmar, east through Thailand, Cambodia, Laos, Vietnam and southern China and southwards through the Malaysian Peninsula and archipelagoes (Gray 1831, Taylor 1963, Manthey 2008, Nguyen et al. 2018, Liu and Rao 2019, Nguyen et al. 2019). They are well-known as the Mountain Horned Dragon or Pricklenape Lizard due to a row of nuchal and dorsal crest scales that extend from neck to tail base (Das 2010). The shape, size and number of the nuchal and dorsal crest scales are diagnostic in *Acanthosaura*: for instance, *Acanthosaura armata* (Hardwick, 1827), *A. capra* (Günther, 1861), *A. cardamomensis* Wood, Grismer, Grismer, Neang, Chav & Holden, 2010, *A. murphyi* Nguyen, Do, Hoang, Nguyen, McCormack, Nguyen, Orlov, Nguyen & Nguyen, 2018, *A. nataliae* Orlov, Truong, & Sang, 2006 and *A. phuketensis* Pauwels, Sumontha, Kunya, Nitikul, Samphanthamit, Wood & Grismer, 2015 are classified as a long-horned group exhibiting the largest nuchal and dorsal spines with lengths greater than 10 mm; *A. bintangensis* Wood, Grismer, Grismer, Northayati, Chan & Bauer, 2009, *A. brachypoda* Ananjeva, Orlov, Nguyen & Ryabov, 2011, *A. crucigera* Boulenger, 1885, *A. lepidogaster* (Cuvier, 1829), *A. phongdienensis* Nguyen, Jin, Vo, Nguyen, Zhou, Che, Murphy, & Zhang, 2019, *A. titiwangsaensis* Wood, Grismer, Grismer, Northayati, Chan & Bauer, 2009 and *A. tongbiguanensis* Liu & Rao, 2019 belong to a short-horned group exhibiting nuchal and dorsal spines that are shorter than 10 mm; and the nuchal crest is absent in *A. coronata* Günther, 1861 (Chan-ard et al. 1999, Pauwels et al. 2002, Pauwels et al. 2003, Ananjeva et al. 2008). However, the spines are not the only diagnostic character and modern morphological and molecular techniques are used as identification tools for classifying these agamid lizards.

The recent morphological and molecular investigations of *Acanthosaura* revealed five species across Thailand: *Acanthosaura lepidogaster* from northern region (Das 2010); *A. cardamomensis* from the eastern region and Cambodia (Wood Jr. et al. 2010); *A. crucigera* from the western region and southern Myanmar (Pauwels et al. 2003, Grismer et al. 2008); *A. phuketensis* from Phuket Island and the south-western region (Pauwels et al. 2015); and *A. armata* from the southern region (Chan-ard et al. 1999, Pauwels et al. 2002). Hence, one-third of *Acanthosaura* species have been reported from Thailand so far.

A recent field sampling of *Acanthosaura* species across the Thanon Thong Chai Mountain Range in Thailand revealed three populations: *A. crucigera*, *A. lepidogaster* and a new population which has a morphology different from the other species. This population exhibits a large yellowish-orange (females) or bright yellow (males) nuchal crest on the dorsum. To clarify the taxonomic status of this *Acanthosaura* population, we describe the new species on the basis of its distinctive morphological and colouration characteristics and a genetic analysis. Its name is provided to be included in conservation planning for agamid species in wildlife sanctuaries and national parks in the Thanon Thong Chai Mountain Range.

## Materials and methods

### Specimen collection

Specimens were collected by hand from two locations in Thanon Thong Chai Mountain Range, Thailand: one specimen from Mae Sariang District, Mae Hong Son Province on 27 April 2018; seven specimens from Sop Khong Subdistrict, Omkoi District, Chiang Mai Province on 14 November 2018; and one specimen from Nang Lae Subdistrict, Mueang District, Chiang Rai Province on 24 January 2019. For all specimens, photographs were taken to document their colour patterns prior to euthanisation. Liver samples were taken and stored in absolute ethanol. Specimens were fixed in 10% formalin before being transferred to 70% ethanol for permanent storage.

Comparative materials of all currently recognised species of *Acanthosaura* in Thailand were examined from the Natural History Museum, National Science Museum, Technopolis, Pathum Thani Province (THNHM) and the Queen Saovabha Memorial Institute, Thai Red Cross Society, Bangkok Province, Thailand (QSMI): *Acanthosaura armata* from Hala-Bala, Narathiwat Province (THNHM15209), Sungai Kolok District, Narathiwat Province (THNHM18884); *Acanthosaura cardamomensis* from Koh Kut, Trat Province (THNHM15597, 20169), Khao Yai National Park, Nakhon Ratchasrima Province (THNHM24711, 24712, 24715); *Acanthosaura crucigera* from Na Yong District, Trang Province (QSMI1594, THNHM28061-28062), Taksin Maharat National Park, Muang District, Tak Province (QSMI1590, 1591, 1592, 1593, THNHM28507, 28508), Thong Pha Phum District, Kanchanaburi Province (THNHM22658), Huai Kha Khaeng, Lan Sak District, Uthai Thani Province (THNHM18594); *Acanthosaura lepidogaster* from Phu Luang District, Loei Province (THNHM08736, 08777), Phu Kieo District, Chaiyaphum Province (THNHM19619), Ban Sun Phae Kae, Chiang Dao District, Chiang Mai Province (THNHM20537), Roi Praputabath, Umphang District, Tak Province (THNHM20647), Huai Na Tee, Pua District, Nan Province (THNHM10080), Doi Khun Tan National Park, Lam Phun Province (THNHM16569, 16570, 16571); *Acanthosaura phuketensis* from Ton Sai Waterfall, Thalang District, Phuket Province (THNHM08865), Khao Sok, Ban Ta Khun, Surat Thani Province (THNHM22663); and *Acanthosaura nataliae* from Xe Sap, Samoy, Lao (THNHM13454, 13455).

In addition, the comparative morphological characters of other taxa in the genus *Acanthosaura* were also taken from original descriptions and subsequent studies (Günther 1861, Boulenger 1885, Ananjeva et al. 2008, Wood Jr. et al. 2009, Wood Jr. et al. 2010, Ananjeva et al. 2011, Nguyen et al. 2018, Pauwels et al. 2015, Nguyen et al. 2019). For species concepts, the combination of morphological species concept and evolutionary species concept were applied to describe genealogical relationships (Wiley and Lieberman 2011).

### Morphological study

Forty-three meristic and measured characters were noted for each specimen of the type series. Measurements were recorded to the nearest 0.01 mm with a Vernier calliper. Measurements were performed on the left side (Wood Jr. et al. 2009, Wood Jr. et al. 2010, Ananjeva et al. 2011). Meristic characters were counted on the left and right sides, respectively. The list and methodology of measurements and meristic counts followed Pauwels et al. (2015): SVL – snout-vent length, measured from the tip of the snout to the tip of the vent; TaL – tail length, measured from the posterior margin of the vent to the tip of the tail; TBW – tail base width, maximum width at tail base; HL – head length, measured from the posterior edge of the rectis of the jaw to the tip of the snout; HW – head width, maximum head width, the width at the level of the tympanum; HD – maximum head height, measured across the parietal region; SL – snout length, measured from the anterior edge of the orbit to the tip of the snout; ORBIT – orbit diameter, measured from the posterior to the anterior edge of the orbit; EYE – eye diameter, measured from the posterior to the anterior edge of the eye; TD – tympanum diameter, measured horizontally from the anterior to the posterior border of the tympanum; TN – scales absent on tympanum (0) or present (1); PS – postorbital spine length, measured from the base to the tip of the spine; NSL – maximum length of the largest spine in the nuchal crest measured from the base to the tip; DS – maximum length of the largest spine in the dorsal crest measured from the base to the tip; WNC – maximum width of the spines in the nuchal crest, measured at the base; DIAS – length of the diastema, measured from the posterior end of the nuchal crest to the anterior end of the dorsal crest; DIASN – number of scales in the vertebral crest scale diastema counted from the posterior end of the nuchal crest to the anterior end of the dorsal crest; FOREL – forelimb length, measured from axilla to the proximal edge of the palmar region; HINDL – hindlimb length, measured from the groin to the proximal edge of the plantar region; SUPRAL – number of supralabials; INFRAL – number of infralabials; VENT – number of ventral scales counted at the midline from the anterior edge of the shoulders to the edge of the vent; FI – number of subdigital lamellae on the fourth finger; TO – subdigital lamellae on the fourth toe; OS – length of the occipital spine, measured from the base to the tip; NSSOS – number of scales surrounding the occipital spine; ; CS – number of canthus rostralis-supraciliary scales, counted from the nasal scale to the posterior end of the ridge at the posterior margin of the orbit; RW – rostral width; RH – rostral height; RS – number of scales bordering the rostral scale; NS – number of scales between the nasals; NCS – number of scales between the fifth canthals; NSCSL – number of scales from the fifth canthal to the fifth supralabial; NR – number of scales between the nasal and the rostral scales; NSSLC – number of scales between the seventh supralabial and the sixth canthal; MW – mental width; MH – mental height; PM – number of scales bordering the mental; YAS – presence (1) or absence (0) of a Y-shaped arrangement of enlarged scales on the snout; ND – presence (1) or absence (0) of a black, diamond shaped, nuchal collar; LKP – presence (1) or absence (0) of a light knee patch; BEP – presence (1) or absence (0) of a black eye patch; ESBO – presence (1) or absence (0) of elliptical scales below the orbit; GP – size of gular pouch scored as absent, small, medium or large; OF – presence (1) or absence (0) of oblique fold anterior to the forelimb insertion.

### Molecular techniques

Molecular data were generated for seven specimens from Thanon Thong Chai Mountain Range, with other *Acanthosaura* taxa and outgroup (*Calotes emma*) obtained from GenBank (Macey et al. 1997a, Macey et al. 2000, Zug et al. 2006, Okajima and Kumazawa 2010, Wood Jr. et al. 2010, Yu et al. 2015) (Table 1). Liver tissues were used for DNA extraction with TIANamp Genomic DNA Kit. The samples were lysed using proteinase K for 3-4 hours at 56ºC. The DNA was eluted from the spin column with 200 µl of buffer.

Polymerase chain reaction (PCR) was prepared using an EP0402 TAQ DNA POLYMERASE. Two primers, METF6 (L4437a; 5’-AAGCTTTCGGGCCCATACC-3’) and ACANTHND2.833. R1 (5’-AGGGAGGTTATTGTTGCTAG-3’), were used to amplify a 698 bp fragment of the NADH dehydrogenase subunit 2 (ND2) gene (Macey et al. 1997b, Kalyabiana-Hauf et al. 2004, Gamble et al. 2008, Wood Jr. et al. 2010). PCR protocol for the amplification of genomic DNA began with an initial denaturation for 2 min at 95ºC, followed by 95ºC for 35 s, annealing at 50ºC for 35 s and extension at 72ºC for 154 s per cycle for 32 cycles (Jackman et al. 2008).

### Phylogenetic analyses

The ND2 sequences were aligned using ClustalW v.1.83 (Thompson et al. 1994) integrated in MEGA6 v.6 (Tamura et al. 2013) with default parameters. All DNA sequences were translated into amino acids to confirm the absence of premature stop codons in the sequences. Average genetic uncorrected distances (pairwise distance) between individuals and mitochondrial clades were calculated in MEGA6 v.6. The Maximum Likelihood analysis (ML) was performed using MEGA6 v.6 with 2000 tree search replicates, 25 initial GAMMA rate categories and final optimisation using four GAMMA shape categories.

Bayesian Interference was performed in Mr.Bayes v.3.1.2 (Ronquist and Huelsenbeck 2003), based on best-fit models of sequence evolution selected by Mr.Modeltest 2.3 (Nylander 2004) under the Akaike Information Criterion (AIC). To calculate Bayesian posterior probabilites (BPP), 2000 pseudo-replicates of the rapid bootstrap algorithm were run for 20 million generations with tree sampling every 100 generations implementing a General Time Reversible model (GTR) and GAMMA distribution of nucleotide rates. Bayesian posterior probabilities were then estimated using a Markov Chain Monte Carlo (MCMC) sampling approach after the average standard deviations once reached 0.002. A 50% majority consensus tree was generated after discarding 20% of initial samples as burn-in. Bootstrap values 70% for ML and BPP of ≥ 95% were considered as they are indicators of strongly-supported nodes (Hillis and Bull 1993, Felsenstein 2004).

## Taxon treatments

### Acanthosaura aurantiacrista sp. n.

212B9A56-ACDD-536A-958C-C3115F384365

urn:lsid:zoobank.org:act:E0D80F05-8884-4426-B2D6-6C939310D2A4

#### Materials

- **Type status:** Holotype. **Location:** country: Thailand (northern region); stateProvince: Mae Hong Son Province; county: Mae Sariang District; verbatimElevation: 728 m; verbatimLatitude: 18°09'02.8"N; verbatimLongitude: 97°58'50.2"E; verbatimCoordinateSystem: degrees minutes seconds; verbatimSRS: WGS84; **Event:** year: 2018; month: April; day: 27; habitat: evergreen forests on hills up to at least 600 m elevation; fieldNotes: collector = Poramad Trivalaira; **Record Level:** institutionCode: THNHM; collectionCode: 28064; basisOfRecord: PreservedSpecimen; dynamicProperties: sex = adult female
- **Type status:** Paratype. **Location:** country: Thailand (northern region); stateProvince: Chiang Mai Province; county: Omkoi District; locality: Sop Khong Subdistrict; verbatimElevation: 935; verbatimLatitude: 17°39'45.4"N; verbatimLongitude: 98°11'53.6"E; verbatimCoordinateSystem: degrees minutes seconds; verbatimSRS: WGS84; **Event:** year: 2018; month: November; day: 14; habitat: evergreen forests on hills up to at least 600 m elevation; fieldNotes: collector = Kirati Kunya; **Record Level:** institutionCode: THNHM; collectionCode: 28521; basisOfRecord: PreservedSpecimen; dynamicProperties: sex = adult female
- **Type status:** Paratype. **Record Level:** institutionCode: THNHM; collectionCode: 28522; basisOfRecord: PreservedSpecimen; dynamicProperties: sex = adult female, same collection date, collector and location as paratype THNHM28521
- **Type status:** Paratype. **Record Level:** institutionCode: THNHM; collectionCode: 28523; basisOfRecord: PreservedSpecimen; dynamicProperties: sex = subadult male, same collection date, collector and location as the THNHM28521
- **Type status:** Paratype. **Record Level:** institutionCode: THNHM; collectionCode: 28524; basisOfRecord: PreservedSpecimen; dynamicProperties: sex = subadult male, same collection date, collector and location as the THNHM28521
- **Type status:** Paratype. **Record Level:** institutionCode: QSMI; collectionCode: 1446; basisOfRecord: PreservedSpecimen; dynamicProperties: sex = adult female, same collection date, collector and location as the THNHM28521
- **Type status:** Paratype. **Record Level:** institutionCode: QSMI; collectionCode: 1447; basisOfRecord: PreservedSpecimen; dynamicProperties: sex = adult female, same collection date, collector and location as the THNHM28521
- **Type status:** Paratype. **Record Level:** institutionCode: QSMI; collectionCode: 1448; basisOfRecord: PreservedSpecimen; dynamicProperties: sex = adult male, same collection date, collector and location as the THNHM28521

#### Molecular analyses

**Figure 1.**
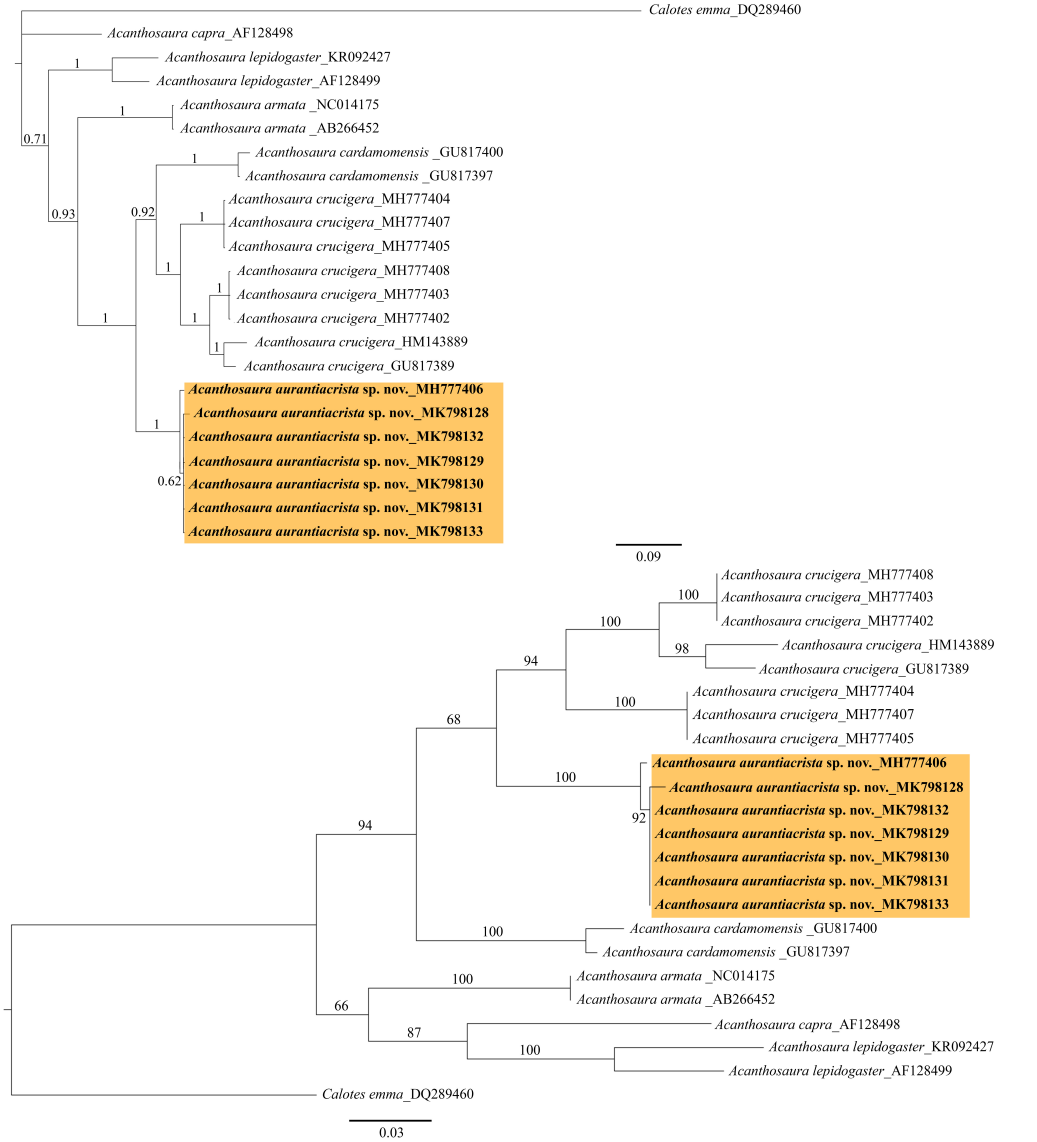
Phylogenetic analysis of the mitochondrial NADH dehydrogenase subunit 2 gene (ND2) of genus *Acanthosaura* and *Calotes emma_*DQ289460. The upper diagram is from the Bayesian analysis; the lower is from the Maximum Likelihood analysis.

Molecular comparisons of 698 nucleotides of the ND2 gene revealed differences of 0-1.2% amongst seven specimens of *Acanthosaura aurantiacrista* sp. n. (GenBank MH777406, MK798128, MK798129, MK798130, MK798131, MK798132 and MK798133) (Table 2). The ND2 gene sequences of the seven specimens of *A. aurantiacrista* sp. n. showed differences of 10.9-14.5% compared to eight specimens of *A. crucigera* (GenBank GU817389, HM143889, MH777402, MH777403, MH777404, MH777405, MH777407 and MH777408); differences of 13.8-15.0% compared to two specimens of *A. cardamomensis* (GenBank GU817397 and GU817400); differences of 16.2-16.3% compared to two specimens of *A. armata* (GenBank AB266452 and NC014175); differences of 18.0-19.6% compared to two specimens of *A. lepidogaster* (GenBank AF128499 and KR092427); and differences of 19.1-19.8% compared to a specimen of *A. capra* (GenBank AF128498) (Table 4). The phylogenetic relationships within the genus *Acanthosaura* revealed through Maximum-Likelihood trees and Bayesian Inference tree of the ND2 gene showed high posterior probabilities and high bootstrap support values (Fig. 1). The *A. aurantiacrista* sp. n. lineage was sister to the *A. crucigera* and *A. cardamomensis* lineage and warrants separate species recognition.

#### Mophological comparison

*Acanthosaura aurantiacrista* sp. n. differs from *A. armata* in presenting fewer INFRAL (9-11 vs. 12-15), more FI (17-23 vs. 13-17), more TO (25-29 vs. 19-26), fewer NS (5-6 vs. 6-10), fewer NCS (11-13 vs. 10-17) and the presence of a BEP and more GP (1-4 vs. 1) (Suppl. material 1).

*Acanthosaura aurantiacrista* sp. n. differs from *A. bintangensis* in presenting greater TaL/SVL ratio (1.40-1.70 vs. 1.30-1.40), greater PS/HL ratio (0.24-0.84 vs. 0.07-0.19), greater DS/HL ratio (0.15-0.38 vs. 0.08-0.09), fewer DIASN (8-9 vs. 11-15), more VENT (63-66 vs. 51-55), fewer NSSOS (5 vs. 6-7), fewer NS (5-6 vs. 8) and the presence of a LKP.

*Acanthosaura aurantiacrista* sp. n. differs from *A. brachypoda* in presenting greater PS/HL ratio (0.24-0.84 vs. 0.11), greater DS/HL ratio (0.15-0.38 vs. 0.06), fewer RS (4-6 vs. 5-9), fewer NS (5-6 vs. 9) and more GP (1-4 vs. 0).

*Acanthosaura aurantiacrista* sp. n. differs from *A. capra* in presenting fewer INFRAL (9-11 vs. 12-13), more FI (17-23 vs. 16-17), more TO (25-29 vs. 22-24), fewer NS (5-6 vs. 9) and the presence of an occipital spine and scales surrounding the occipital spine.

*Acanthosaura aurantiacrista* sp. n. differs from *A. cardamomensis* in presenting fewer RS (4-6 vs. 7-9), fewer NS (5-6 vs. 7-10) and fewer NSSLC (9-13 vs. 10-19).

*Acanthosaura aurantiacrista* sp. n. differs from *A. coronata* in presenting greater TaL/SVL ratio (1.40-1.70 vs. 0.60-1.00), more FI (17-23 vs. 13-14), more TO (25-29 vs. 17-19), fewer RS (4-6 vs. 9), fewer NS (5-6 vs. 7-9), fewer NR (1-2 vs. 3-4) and the presence of a postorbital spine, nuchal spine, dorsal spine, diastema, occipital spine, YAS, ND, BEP and more GP (1-4 vs. 0).

*Acanthosaura aurantiacrista* sp. n. differs from *A. crucigera* in presenting fewer DIASN (8-9 vs. 9-25), more VENT (63-66 vs. 55-63), more FI (17-23 vs. 16-18), more TO (25-29 vs. 21-26), fewer RS (4-6 vs. 7-8), fewer NS (5-6 vs. 7-9) and more GP (1-4 vs. 1-2).

*Acanthosaura aurantiacrista* sp. n. differs from *A. lepidogaster* in presenting greater TaL/SVL ratio (1.40-1.70 vs. 1.00-1.50), greater PS/HL ratio (0.24-0.84 vs. 0.06-0.17), greater NSL/HL ratio (0.35-0.95 vs. 0.12-0.15), greater DS/HL ratio (0.15-0.38 vs. 0.06-0.15), fewer DIASN (8-9 vs. 10-14), more VENT (63-66 vs. 52-61), more TO (25-29 vs. 22-23), greater OS/HL ratio (0.19-0.44 vs. 0.14-0.15), fewer NS (5-6 vs. 7-8), fewer PM (4 vs. 5), absence of scale on tympanum and more GP (1-4 vs. 0-1).

*Acanthosaura aurantiacrista* sp. n. differs from *A. murphyi* in presenting greater PS/HL ratio (0.24-0.84 vs. 0.16-0.34), greater NSL/HL ratio (0.35-0.95 vs. 0.24-0.43), fewer INFRAL (9-11 vs. 12-14), more FI (17-23 vs. 15-18), more TO (25-29 vs. 21-23), fewer RS (4-6 vs. 8-9), fewer NS (5-6 vs. 7-8), fewer NR (1-2 vs. 3-4) and the absence of a tympanum scale.

*Acanthosaura aurantiacrista* sp. n. differs from *A. nataliae* in presenting fewer VENT (63-66 vs. 64-71), fewer RS (4-6 vs. 7), fewer NSSLC (9-13 vs. 13-16) and the presence of an occipital spine, scales surrounding the occipital spine, ND and LKP.

*Acanthosaura aurantiacrista* sp. n. differs from *A. phongdienensis* in presenting greater PS/HL ratio (0.24-0.84 vs. 0.06-0.09), greater NSL/HL ratio (0.35-0.95 vs. 0.07-0.18), larger DS/HL ratio (0.15-0.38 vs. 0.03-0.07), more FI (17-23 vs. 14-17), more TO (25-29 vs. 19-23) and the presence of a diastema.

*Acanthosaura aurantiacrista* sp. n. differs from *A. phuketensis* in presenting greater NSL/HL ratio (0.35-0.95 vs. 0.21-0.39), fewer DIASN (8-9 vs. 12-17), more FI (17-23 vs. 15-17), more TO (25-29 vs. 21-24), fewer RS (4-6 vs. 5-9), fewer NS (5-6 vs. 7-8) and more GP (1-4 vs. 1-2).

*Acanthosaura aurantiacrista* sp. n. differs from *A. titiwangsaensis* in presenting greater PS/HL ratio (0.24-0.84 vs. 0.14-0.18), greater NSL/HL ratio (0.35-0.95 vs. 0.11-0.18), greater DS/HL ratio (0.15-0.38 vs. 0.07-0.09), fewer DIASN (8-9 vs. 10-13), more VENT (63-66 vs. 47-57), fewer NS (5-6 vs. 8) and the presence of a LKP.

*Acanthosaura aurantiacrista* sp. n. differs from *A. tongbiguanensis* in presenting greater PS/HL ratio (0.24-0.84 vs. 0.13-0.19), greater NSL/HL ratio (0.35-0.95 vs. 0.15-0.21), greater OS/HL ratio (0.19-0.44 vs. 0.16-0.23), fewer RS (4-6 vs. 6-9), fewer NS (5-6 vs. 8-9) and more GP (1-4 vs. 1-2).

##### Diagnosis

*Acanthosaura aurantiacrista* **sp. n.** is differentiated from all other congeners by this combination of characters: A large size (maximum SVL 130.1 mm for males and 119.3 mm for females) and a single long conical spine above the posterior margin of the eye; a large spine on the occiput between the tympanum and nuchal crest; tympanum naked, large, roundish; large developed gular pouch; scales on flanks randomly intermixed with small keeled and small tubercle scales; large nuchal crest with 8 large dagger-like and pointed spines; narrow diastema with 8-9 scales between the nuchal and vertebral crests; vertebral crest composed of large dagger-like, pointed spines beginning at the shoulder region and decreasing in size until the base of the tail; nuchal and dorsal crests are orange in females and yellow in males; tail 1.40-1.70 times the SVL; and black collar and black eye patch present, extending posteriorly until reaching the nuchal crest.

#### Description

**Figure 2.**
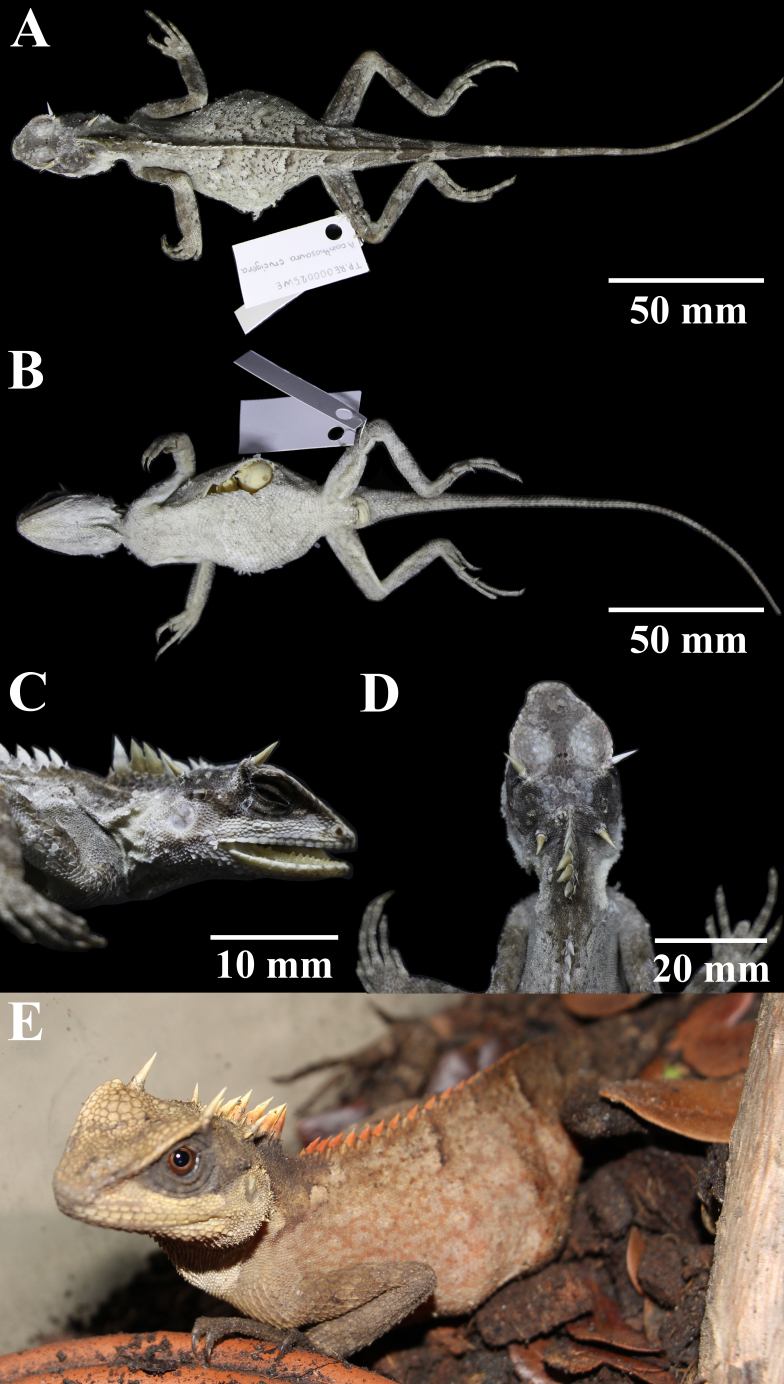
Holotype THNHM28064 of *Acanthosaura aurantiacrista* sp. n., adult female from Mae Sariang District, Mae Hong Son Province, Thailand. **A.** Dorsal and **B.** Ventral views of the body; **C.** Lateral and **D.** Dorsal view of the head; **E.** In life.

**Description of the holotype**: Adult female. SVL 105.7 mm; TaL 151.8 mm (1.44 times SVL), tail complete; HL (23.3 mm) slightly longer than HW (18.9 mm); HL one-fifth SVL (0.22 times SVL), HW narrow (0.179 times SVL) and HD tall (0.64 times HL); head triangular in dorsal and lateral views; SL moderately long (0.46 times HL); RW wide (2.31 times RH); steeply sloping anteriorly; CS prominent, forming a large projecting shelf extending above eye, composed of 14/13 large scales; shelf terminates with a notch anterior to postorbital spine; rostrum moderate in size, rectangular, bordered laterally by first SUPRALs and posteriorly by five smaller scales; nostrils roundish, surrounded by one prenasal anteriorly, four postnasals posteriorly and two subnasals; six NS; oval supranasals; large scales above orbit weakly keeled; three rows of moderately-keeled scales below orbit extending from the anterior margin of the eye to posterior; large EYE (0.28 times HL) and ORBIT (0.44 times HL); interorbital, prefrontal and frontal scales slightly keeled and smaller than scales below orbit; seven large, keeled, azygous prefrontal scales arranged in a Y-shaped pattern; parietal eyespot surrounded by a larger row of scales; large conical PS above posterior margin of the eye surrounded by five small lanceolate scales; single row of seven large keeled scales extending from suborbital below posterior margin of eye to above tympanic margin, increasing in size posteriorly; elongated conical OS on lateral margin of nape surrounded by a rosette of five small lanceolate NSSOS; tympanum exposed, roundish, with a size two-thirds that of EYE (0.69 times EYE), surrounded by tiny conical scales; thirteen rectangular SUPRALs similar in size; mental pentagonal, larger than adjacent INFRALs; two postmentals similar in size, four scales contacting PM; chin shields large, extending posteriorly to angle of jaw, separated from infralabials by one scale row anteriorly and three at angle of jaw; eleven rectangular INFRALs of similar size; gular scales sharply keeled and spinose with a larger midventral row; extensible dewlap present; nuchal crest composed of eight elongated, dagger-like scales, bordered on each side by two rows of large, flat, keeled, triangular scales; nuchal crest followed by a diastema of nine DIASN at base of nape; dorsal body crest extending from posterior margin of diastema to base of tail; dorsal crest composed of small laterally compressed, triangular epidermal scales, bordered by a row of smaller paravertebral triangular scales; DS slightly decreasing to sacrum, then fading progressively; and nuchal and dorsal crests present as orange in live specimen (Fig. 2).

Body robust, triangular in cross-section; dorsal body scales small, mixed with some large-keeled scales without a regular pattern, keels projecting posteriorly; pectoral and abdominal scales larger than DS; keeled, semi-transverse rows arranged; keeled scales anterior to large vent; limbs relatively long, dorsal forelimb and hindlimb scales keeled and larger than VENT; five digits on manus; subdigital scales keeled, FI 23/21; five digits on pes; subdigital scales keeled, TO 27/26; TaL 1.44 times SVL, tail covered with keeled spinose scales, keels on subcaudals directed posteriorly; subcaudals much longer than supracaudals; TBW 10.3 mm; and four white creamy eggs with a diameter of approximately 10 mm inside the body.

##### Variation

**Figure 3.**
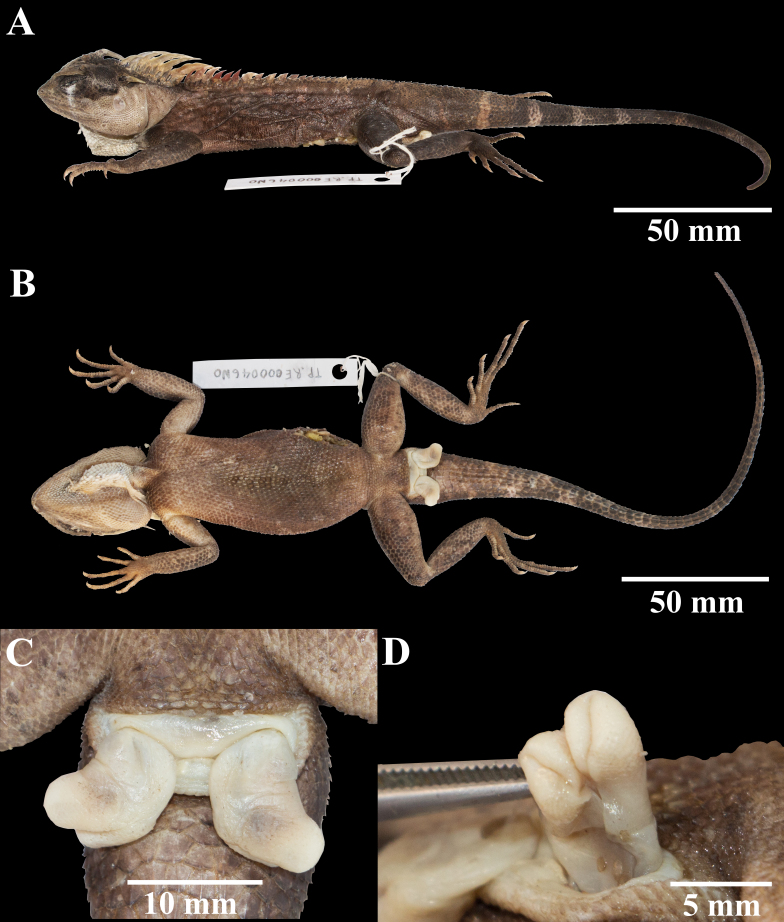
Paratype QSMI1448 of *Acanthosaura aurantiacrista* sp. n., adult male from Sop Khong Subdistrict, Omkoi District, Chiang Mai Province, Thailand. **A.** Dorsal and **B.** Ventral views of body; **C.** Cloaca opening with everted hemipenis; **D.** Everted left hemipenis.

The female paratypes resemble the holotype in all aspects, and the observed differences were a larger PS (7-10.5 vs. 5.7) and OS (5.3-7.5 vs. 4.5) and greater NSL (9.6-12.7 vs. 8.3), wider WNC (0.9-1.6 vs. 0.6), more SUPRAL (10-11/10 vs. 13/13) and fewer number of FI (18-20/17-20 vs. 23/21), higher number of NSSLC (10 vs. 13) in QSMI1446, QSMI1447, THNHM28521 and THNHM28522, and larger GP (3 vs. 1) in QSMI1446 and THNHM28522. The adult male paratype (QSMI1448) differs from the adult female holotype in presenting a longer SVL (130.1 vs. 105.7), greater TaL (202.2 vs. 151.8) and SL (22.3 vs. 10.7), wider TBW (19.2 vs. 10.3), greater HD (21.7 vs. 14.9), larger ORBIT (8.5 vs. 6.4), longer PS (19.1 vs. 5.7), OS (10 vs. 4.5), greater NSL (21.6 vs. 8.3), FOREL (54.2 vs. 49.8), HINDL (71.4 vs. 59.7) and GP (4 vs. 1) size, wider WNC (2.9 vs. 0.6), narrower DIAS (3.5 vs. 5.4) and fewer number of SUPRAL (10 vs. 13) and FI (19/18 vs. 23/21). Two subadult male paratypes are smaller and present fewer differences in morphological characters compared with the holotype, except for a longer PS (5.5-7.5 vs. 5.7) and OS (5.9 vs. 4.5) in THNHM28523, a greater WNC (0.7-0.8 vs. 0.6) in THNHM28523 and THNHM28524 and higher number of TO (29/29 vs. 27/26) (Fig. 3). Morphometric and meristic data for the type series are shown in Table 3.

##### Coloration in life

**Figure 4.**
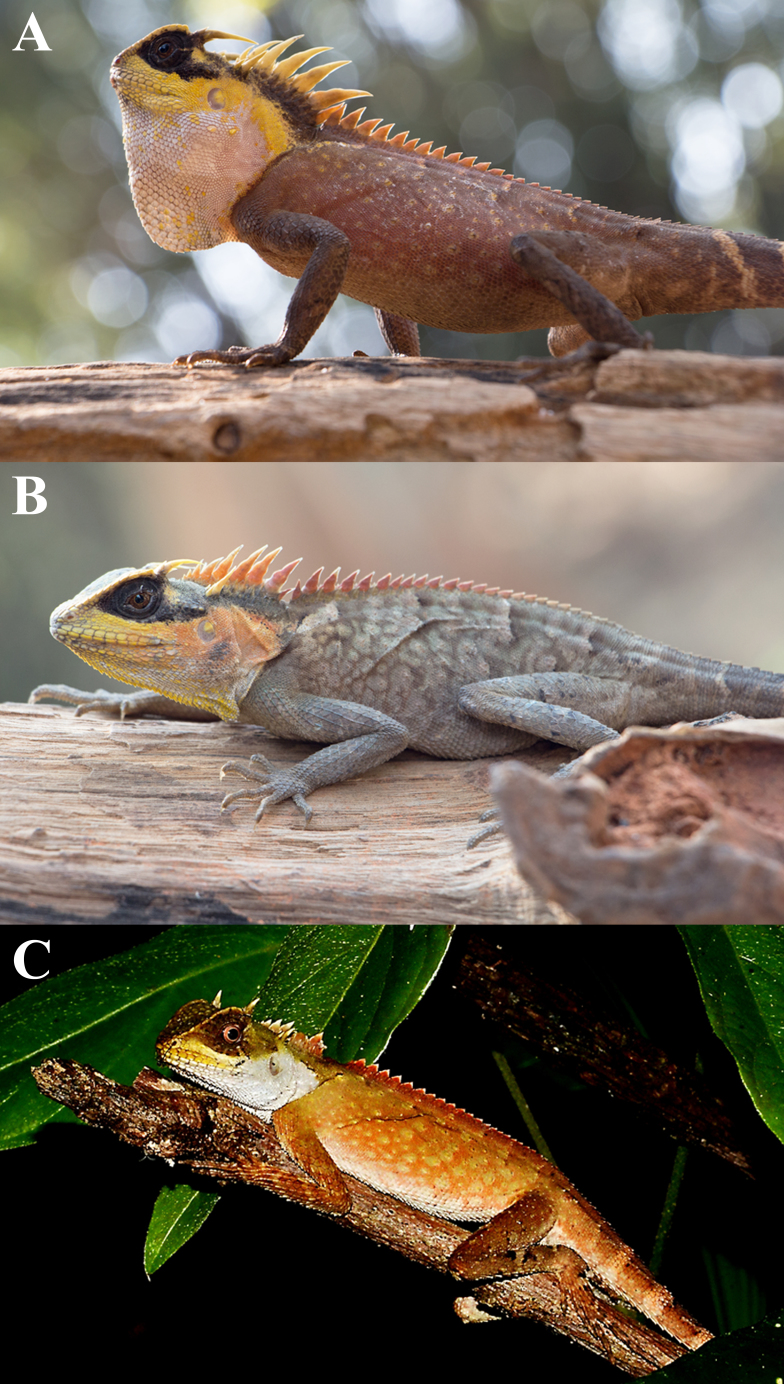
Morphology of *Acanthosaura aurantiacrista* sp. n. from Sop Khong Subdistrict, Omkoi District, Chiang Mai Province, Thailand. **A.** Adult male paratype QSMI1448; **B.** Adult female paratype THNHM28522; **C.** Subadult male paratype THNHM28523.

Males – Front of head dark with brownish-yellow colouration on the canthals; lips yellow; black eye patch; lateral head and neck yellow; gular pouch white; postorbital and occipital spines creamy yellow; nuchal crest bright yellow with some orange; dorsal crest orange-yellow; body rusty-brown with some brownish-yellow keeled scales; whitish-yellow ocellated spot at the knee and elbow; ventral creamy white with some dark spots or dirty brown colouration on the abdomen with creamy brown colouration on pectoral and forelimbs; forelimbs and hindlimbs dark brown dorsally; tail banded with dark brown and dirty light brown (Fig. 4).

In addition, the hemipenis of QSMI1448 is everted approximately 10 mm from the cloaca opening to the hemipenis tip on each side. The hemipenis on each side diverged to a symmetrical spongy millet shape with a width of approximately 5 mm. In preserved ethanol, the hemipenes exhibited a creamy yellow colouration.

Females - Front of head whitish-yellow or creamy white; lips orange-yellow; black eye patch; lateral head and neck yellow intermixed with orange; gular pouch white or yellow intermixed with white; postorbital and occipital spines creamy yellow; nuchal crest yellowish-orange or reddish-orange; dorsal crest reddish-orange; body with brownish-grey or rusty-grey marbled reticulum with some grey keeled scales; whitish or whitish-yellow ocellated spot at the knee and elbow, with certain others indicated on the forelimbs and hindlimbs; ventral creamy white or creamy yellow intermixed with some dark brown on the abdomen, ventral region of forelimbs and hindlimbs; forelimbs and hindlimbs brownish-grey dorsally; tail banded with dark brown and dirty light brown.

##### Etymology

The specific epithet *aurantiacrista* came from a combination of the Latin words aurantiaco (orange) and crista (crest). The name refers to a distinctive characteristic of the first discovered female specimen, which exhibited nuchal and dorsal crests with an orange colour. We suggest the following common names: kingkakhaownaam seesom (Thai), orange crested horned lizard (English), orange-verzierter gehörnter Nackenstachler (German) and Acanthosaurus à crête orange (French).

#### Distribution

**Figure 5.**
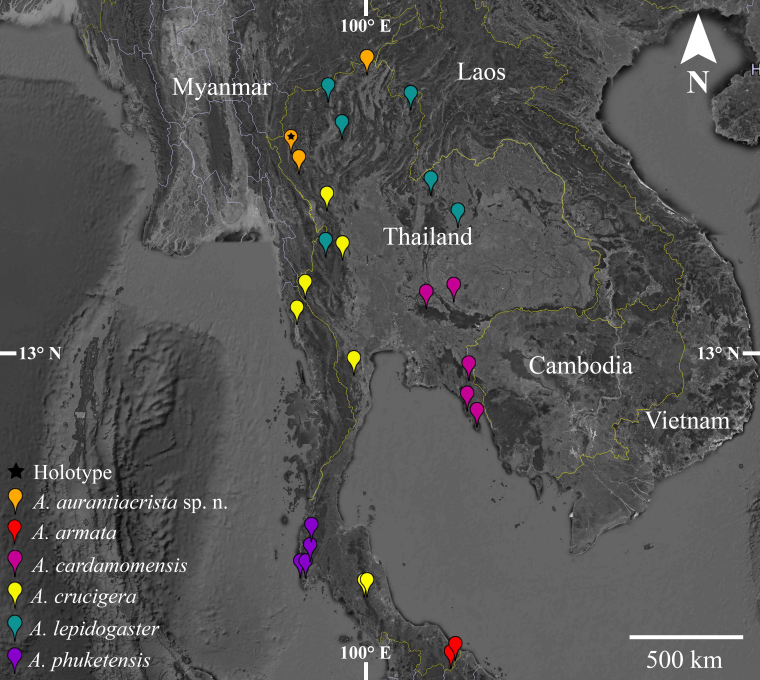
Distribution of *Acanthosaura* species in Thailand.

*Acanthosaura aurantiacrista* sp. n. occurs in the Thanon Thong Chai Mountain Range in northern Thailand: Mae Sariang District, Mae Hong Son Province (18°14'54.8"N, 97°98'38.2"E) at 728 m a.s.l.; Sop Khong Subdistrict, Omkoi District, Chiang Mai Province (17°63'55.4"N, 98°18'15.6"E) at 935 m a.s.l.; and Nang Lae Subdistrict, Mueang District, Chiang Rai Province (20°02'35.3"N, 99°54'12.1"E) at 636 m a.s.l. This species usually lives in rainforests on mountains at elevations over 600 m a.s.l. (Fig. 5).

#### Ecology

**Figure 6.**
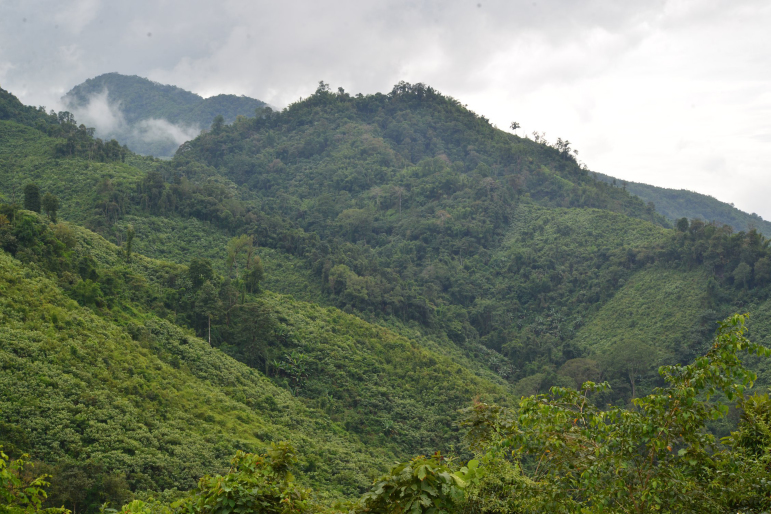
Ecological area of *Acanthosaura aurantiacrista* sp. n. from Sop Khong Subdistrict, Omkoi District, Chiang Mai Province, Thailand.

*Acanthosaura aurantiacrista* sp. n. has been found in evergreen forests on hills up to at least 600 m elevation (Fig. 6). It is active during the day on the ground, logs or rocks or 1-2 m above the ground on trees. During night, it is inactive and sleeps on twigs or trees 1-2 m above the ground. This species displays a defence mechanism against approach or provocation consisting of falling to the ground and running away to find refuge under rocks, log hollows or shrubs.

## Discussion

The comparisons revealed morphological characters of a new long-horned lizard species, *Acanthosaura aurantiacrista* sp. n., that were clearly distinct from those of other recognised *Acanthosaura* species, especially the two short-horned lizards, *A. crucigera* and *A. lepidogaster*, whose geographic distributions overlap with that of *A. aurantiacrista* sp. n. in the northern region of Thailand (Cuvier 1829, Boulenger 1885, Grismer et al. 2006, Orlov et al. 2006, Stuart et al. 2006, Ananjeva et al. 2008, Wood Jr. et al. 2009, Wood Jr. et al. 2010, Pauwels et al. 2015). In addition, the molecular analysis indicated that *A. aurantiacrista* sp. n. is closely related to *A. crucigera* and geographically-distant species *A. cardamomensis* (Wood *et al*. 2010). This herpetological discovery yields the sixth member of the genus *Acanthosaura* from Thailand and the taxa in this genus that have not yet been identified, will be described in the near future.

Many forest areas in northern regions of Thailand are currently faced with deforestation due to timber logging and forest fire for human use, which negatively influences the conservation of the herpetological fauna (Delang 2011). However, *Acanthosaura aurantiacrista* sp. n. is expected to distribute throughout the Thanon Thong Chai Mountain Range, which includes several important protected areas where the biological fauna is preserved in northern regions of Thailand, such as Doi Inthanon National Park; Doi Suthep-Pui National Park; Khun Khan National Park; Mae Ngao National Park; Mae Ping National Park; Mae Tho National Park; Mae Wang National Park; Namtok Mae Surin National Park; Op Luang National Park; Op Khan National Park; Chiang Dao Wildlife Sanctuary; Lum Nam Pai Wildlife Sanctuary; Mae Lao-Mae Sae Wildlife Sanctuary; Mae Tuen Wildlife Sanctuary; and Om Koi Wildlife Sanctuary. Moreover, our research team recommends this new long-horned species, whose nuchal and dorsal crests have an appearance similar to fire, as a representative animal for drawing the attention of the local people, scientific community and government towards addressing the current problem of forest burning in northern regions of Thailand.

## Supplementary Material

6CF57014-D7C4-5583-91CF-B24FDE50E476 10.3897/BDJ.8.e48587.suppl1 Supplementary material 1 Comparison of morphometric (in mm) and meristic data for all currently recognised species of *Acanthosaura* and *Acanthosaura aurantiacrista* sp. n., “?” = data not available. Data type morphological Brief description The comparison table of morphometric for *Acanthosaura* lizard File: oo_395107.docx https://binary.pensoft.net/file/395107 Poramad Trivalairat

XML Treatment for Acanthosaura aurantiacrista

## Figures and Tables

**Table 1.** GenBank accession numbers for ND2 sequence and catalogue number of vouchers used in phylogenetic analyses of *Acanthosaura*.

**Taxon**	**Voucher**	**GenBank**	**Locality**
Ingroup			
*Acanthosaura aurantiacrista* sp. n.	THNHM28064	MH777406	Mae Sariang, Mae Hong Son, Thailand
*Acanthosaura aurantiacrista* sp. n.	QSMI1446	MK798128	Sop Khong, Omkoi, Chiang Mai, Thailand
*Acanthosaura aurantiacrista* sp. n.	THNHM28521	MK798129	Sop Khong, Omkoi, Chiang Mai, Thailand
*Acanthosaura aurantiacrista* sp. n.	THNHM28522	MK798130	Sop Khong, Omkoi, Chiang Mai, Thailand
*Acanthosaura aurantiacrista* sp. n.	QSMI1447	MK798131	Sop Khong, Omkoi, Chiang Mai, Thailand
*Acanthosaura aurantiacrista* sp. n.	QSMI1448	MK798132	Sop Khong, Omkoi, Chiang Mai, Thailand
*Acanthosaura aurantiacrista* sp. n.	THNHM28523	MK798133	Sop Khong, Omkoi, Chiang Mai, Thailand
*Acanthosaura crucigera*	QSMI1594	MH777404	Na Yong, Trang, Thailand
*Acanthosaura crucigera*	THNHM28062	MH777405	Na Yong, Trang, Thailand
*Acanthosaura crucigera*	THNHM28061	MH777407	Na Yong, Trang, Thailand
*Acanthosaura crucigera*	CAS229582	GU817389	Kawthaung, Tanintharyi, Myanmar
*Acanthosaura crucigera*	THNHM28057	MH777402	Muang, Tak, Thailand
*Acanthosaura crucigera*	QSMI1593	MH777403	Muang, Tak, Thailand
*Acanthosaura crucigera*	QSMI1592	MH777408	Muang, Tak, Thailand
*Acanthosaura crucigera*	CUMZR2008.05.26.1	HM143889	Petchaburi, Thailand
*Acanthosaura armata*	NSMT-H4595	AB266452	Asia
*Acanthosaura armata*	-	NC_014175	Asia
*Acanthosaura capra*	MVZ222130	AF128498	Vietnam
*Acanthosaura cardamomensis*	FMNH263225	GU817397	Kampot, Cambodia
*Acanthosaura cardamomensis*	FMNH263261	GU817400	Kampot, Cambodia
*Acanthosaura lepidogaster*	MVZ224090	AF128499	Vinh Thu, Vietnam
*Acanthosaura lepidogaster*	MD001	KR092427	Hainan, China
Outgroup			
*Calotes emma*	CAS223062	DQ289460	Rakhine State, Myanmar

**Table 2.** Pairwise distance values (percentages) of the ND2 gene within and amongst six species of the genus *Acanthosaura* and the outgroup *Calotes emma*.

**Taxon**	**1**	**2**	**3**	**4**	**5**	**6**	**7**
*Calotes emma*	-						
*Acanthosaura armata*	33.2	0					
*Acanthosaura cardamomensis*	34.4-35.4	16.5-17.2	1.7				
*Acanthosaura aurantiacrista* sp. n.	31.6-32.6	16.2-16.3	13.8-15.0	0-1.2			
*Acanthosaura crucigera*	34.8-36.7	18.9-21.5	14.0-15.5	10.9-14.5	0-10.9		
*Acanthosaura capra*	34.4	17.3	19.9-21.0	19.1-19.8	21.5-23.3	-	
*Acanthosaura lepidogaster*	36.3	18.2-18.9	18.2-22.2	18.0-19.6	19.9-23.7	16.3-18.5	9.2

**Table 3.** Morphometrical (in mm) and meristic data for the type series of *Acanthosaura aurantiacrista* sp. n. For character abbreviations, see materials and methods. Paired meristic characters are given left/right. NA = not applicable.

	Holotype THNHM 28064 Adult female	Paratype QSMI 1446 Adult female	Paratype THNHM 28521 Adult female	Paratype THNHM 28522 Adult female	Paratype QSMI 1447 Adult female	Paratype QSMI 1448 Adult male	Paratype THNHM 28523 Subadult male	Paratype THNHM 28524 Subadult male
SVL	105.7	118.6	119.3	113.8	105	130.1	85.5	80.8
Tal	151.8	163.8	187	173.8	157.5	202.2	139.9	137
Tal/SVL	1.44	1.38	1.57	1.53	1.5	1.55	1.64	1.7
TBW	10.3	11.3	11	10.8	10	19.2	8.9	7.3
HL	23.3	22.4	24.2	20.4	22.6	22.7	16	15.6
HW	18.9	19.7	19.5	19.9	18.3	19.4	15.6	14.7
HD	14.9	18.4	16.6	15.6	19	21.7	13.1	12.5
SL	10.7	9.1	12.4	10	11.8	12.4	6.6	9.1
ORBIT	10.3	11.1	9.9	10	10.4	11.8	6.8	7.3
EYE	6.4	6.3	6.8	7.5	5.9	8.5	4.4	4.8
TD	4.4	3.6	4.3	4.4	4	4.9	2	3
TD/HD	0.30	0.2	0.26	0.28	0.21	0.23	0.15	0.24
TN	0	0	0	0	0	0	0	0
PS	5.7	8.5	10.5	9.4	7	19.1	7.5	5.5
PS/HL	0.24	0.38	0.43	0.46	0.31	0.84	0.47	0.35
NSL	8.3	9.8	12.7	9.6	11	21.6	7.3	5.5
NSL/HL	0.36	0.44	0.53	0.47	0.49	0.95	0.46	0.35
DS	4.2	4.4	6.5	4.9	5.6	8.7	3.6	2.4
DS/HL	0.18	0.2	0.27	0.24	0.25	0.38	0.23	0.15
WNC	0.6	0.9	1.6	1.2	1.5	2.9	0.8	0.7
DIAS	5.4	4.6	5.2	3.8	4	3.5	3.3	3.5
DIAS/SVL	0.05	0.04	0.04	0.03	0.04	0.03	0.04	0.04
DIASN	9	9	9	8	8	8	8	8
FOREL	49.8/50.2	51.5/51.2	52.7/53.1	45.5/47.1	44.6/44.8	54.2/52.7	37.9/37.8	36.8/36.9
HINDL	59.7/58.4	64.2/60.9	70.7/71.9	58.6/58.5	57.7/59.4	71.4/72.9	48.4/47.9	47.9/46.2
SUPRAL	13/13	10/10	10/10	10/10	11/NA	10/10	10/10	10/10
INFRAL	11/11	10/11	10/10	11/11	11/10	10/9	10/10	11/10
VENT	66	63	66	63	65	66	63	63
FI	23/21	20/20	19/20	18/17	19/19	19/18	18/18	19/19
TO	27/26	26/26	26/27	26/26	26/25	26/27	25/26	29/29
OS	4.5	5.3	6.8	7.5	6.5	10	5.9	3.2
OS/HL	0.19	0.24	0.28	0.37	0.29	0.44	0.37	0.21
NSSOS	5/5	5/5	5/5	5/5	5/5	5/5	5/5	5/5
CS	14/13	11/11	12/12	11/11	10/10	12/11	10/10	11/11
RW	3	3.5	3.6	3.7	3	3.5	2.5	2.5
RH	1.3	1.4	2.1	1.6	1.6	1.6	0.9	1
RS	5	6	6	6	4	5	4	4
NS	6	6	6	6	5	6	6	6
NCS	13	11	11	13	12	13	12	12
NSCSL	8	7	7	7	7	10	6	8
NR	2	1	1	1	1	2	1	1
NSSLC	13	10	10	10	10	12	9	10
MW	1.1	1.2	2.1	1.5	1.1	2.5	2	1.1
MH	1.6	1	1.6	1.1	0.9	1.2	1.5	0.8
PM	4	4	4	4	4	4	4	4
YAS	1	1	1	1	1	1	1	1
ND	1	1	1	1	1	1	1	1
LKP	1	1	1	1	1	1	1	1
BEP	1	1	1	1	1	1	1	1
ESBO	0	0	0	0	0	0	0	0
GP	1	3	2	3	1	4	1	1
OF	1	1	1	1	1	1	1	1
