# Supplementary material for: Acanthosaura aurantiacrista (Squamata: Agamidae), a new long horn lizard from northern Thailand
Source: Biodivers Data J. 2020 May 15;8:e48587. doi: 10.3897/BDJ.8.e48587 (PMC7244599; doi:10.3897/BDJ.8.e48587)
Supplement: Supplementary material 1 — Comparison of morphometric (in mm) and meristic data for all currently recognised species of Acanthosaura and Acanthosaura aurantiacrista sp. n., “?” = data not available. [file bdj-08-e48587-s001.docx]

**TABLE 3.** Comparison of morphometric (in mm) and meristic data for all currently recognized species of *Acanthosaura* and *Acanthosaura aurantiacrista* **sp. n.**, “?” = data not available.

|  | *A. aurantiacrista*  **sp. n.** | *A. armata* | *A. bintangensis* | *A. brachypoda* | *A. capra* | *A cardamomensis* | *A. coronata* | *A. crucigera* | *A. lepidogaster* | *A. murphyi* | *A. nataliae* | *A. phongdienensis* | *A. phuketensis* | *A. tongbiguanensis* | *A. titiwangsaensis* |
| --- | --- | --- | --- | --- | --- | --- | --- | --- | --- | --- | --- | --- | --- | --- | --- |
| SVL | 80.8-130.1 | 69.2-138.0 | 83.9-142.0 | 117.0 | 94.0-137.9 | 82.0-149.0 | 66.0- 86.1 | 69.2-127.0 | 76.5-101.1 | 103.7-127.3 | 106.7-158.0 | 58.5-77.4 | 69.2- 123.5 | 93.0-115.6 | 91.8-118.4 |
| TaL | 137.0-202.2 | 96.6-190.0 | 112.8-206.0 | 185.4 | 133.6-182.1 | 103.0-188.0 | 86.3-105.0 | 130.0-174.0 | 130.6-144.1 | 159.3-195.8 | 132.5-190.0 | 94.6-137.2 | 107.0-205.6 | 144.9-205.0 | 136.0-174.0 |
| Tal/SVL | 1.4-1.7 | 1.2-1.6 | 1.3-1.4 | 1.58 | 1.2-1.5 | 1.2-1.6 | 0.6-1.0 | 1.1-1.8 | 1.6-1.9 | 1.48-1.54 | 1.0-1.5 | 1.5-1.9 | 1.4-1.7 | 1.56-1.85 | 1.1-1.5 |
| TBW | 7.3-19.2 | 15.1-15.6 | ? | ? | ? | 5.8-12.8 | ? | 7.4-14.5 | 5.9-11.8 | ? | 15.0-16.3 | ? | 5.4-14.5 | ? | ? |
| HL | 15.6-24.2 | 6.6-33.7 | 16.9-25.4 | 30.3 | 16.3-38.9 | 16.3-42.2 | 14.4-16.3 | 18.7-23.6 | 18.9-29.7 | 29.1-36.8 | 15.0-43.6 | 18.6-23.8 | 19.7-31.4 | 27.5-33.2 | 20.0-24.3 |
| HW | 14.7-19.9 | 15.3-23.0 | 17.5-23.4 | 20.6 | 16.8-27.0 | 16.4-27.7 | 13.6-17.5 | 16.0-22.3 | 13.4-20.8 | 20.3-24.6 | 20.2-27.8 | 13.1-15.9 | 14.4-22.8 | 18.6-23.3 | 17.5-23.4 |
| HD | 12.5-21.7 | 12.2-18.9 | 15.0-19.2 | 17.2 | 14.8-24.3 | 12.6-21.7 | 11.9-16.8 | 15.7-22.5 | 12.0-12.5 | 18.5-20.6 | 16.9-24.9 | 10.4-13.6 | 10.9-18.6 | 13.9-17.4 | 15.7-20.2 |
| SL | 6.6-12.4 | 6.3-16.6 | 7.9-11.3 | 12.2 | 7.6-16.6 | 8.6-18.7 | 6.9-8.4 | 8.7-12.1 | 9.3-10.2 | 10.3-15.3 | 12.0-19.9 | ? | 6.8-11.0 | 9.2-11.0 | 9.7-12.5 |
| ORBIT | 6.8-11.8 | 5.4-13.3 | 8.4-12.6 | 8.3 | 7.6-11.6 | 5.8-12.7 | 6.9-7.5 | 8.9-10.8 | 4.7-9.1 | 9.9-12.3 | 7.2-10.9 | ? | 6.6-11.2 | 7.7-11.0 | 9.8-13.2 |
| EYE | 4.4-8.5 | 8.0-9.9 | ? | ? | ? | 4.0-8.8 | ? | 3.5-7.2 | 3.2-6.0 | ? | ? | ? | 3.3-7.5 | ? | ? |
| TD | 2.0-4.9 | 2.4-5.2 | 2.5-3.0 | 3.6 | 3.4-5.2 | 2.5-5.8 | 1.7-2.8 | 2.5-3.9 | 2.2-3.0 | 3.2-5.2 | 3.9-7.0 | 1.78-2.81 | 3.5-4.7 | 3.2-4.2 | 2.7-4.0 |
| TD/HD | 0.15-0.30 | 0.19-0.28 | 0.16 | 0.21 | 0.21-0.23 | 0.20-0.27 | 0.14-0.17 | 0.14-0.21 | 0.18-0.24 | 0.17-0.28 | 0.23-0.28 | 0.17-0.22 | 0.22-0.33 | 0.21-0.24 | 0.17-0.20 |
| TN | 0 | 0 | 0 | 0 | 0 | 0 | 0 | 0 | 0-1 | 1 | 0 | 0 | 0 | 0 | 0 |

…...*continued on the next page*

**TABLE 3.** (Continued)

|  | *A. aurantiacrista*  **sp. n.** | *A. armata* | *A. bintangensis* | *A. brachypoda* | *A. capra* | *A cardamomensis* | *A. coronata* | *A. crucigera* | *A. lepidogaster* | *A. murphyi* | *A. nataliae* | *A. phongdienensis* | *A. phuketensis* | *A. tongbiguanensis* | *A. titiwangsaensis* |
| --- | --- | --- | --- | --- | --- | --- | --- | --- | --- | --- | --- | --- | --- | --- | --- |
| PS | 5.5 -19.1 | 4.9-12.0 | 1.9-4.2 | 3.2 | 5.2-10.2 | 3.2-12.7 | Absent | 1.9-7.8 | 1.2-2.5 | 5.6-11.8 | 7.7-17.8 | 1.18-2.07 | 4.6-11.8 | 3.6-6.3 | 3.3-4.4 |
| PS/HL | 0.24-0.84 | 0.22-0.58 | 0.07-0.19 | 0.11 | 0.36 | 0.14-0.45 | 0 | 0.09-0.33 | 0.06-0.17 | 0.16-0.34 | 0.36-0.52 | 0.06-0.09 | 0.23-0.38 | 0.13-0.19 | 0.14-0.18 |
| NSL | 5.5-21.6 | 5.5-11.2 | 1.3-4.7 | 4.7 | 4.2-14.7 | 3.8-17.4 | Absent | 3.1-8.9 | 2.9-3.4 | 7.0-14.9 | 8.5-23.8 | 1.24-4.18 | 4.1-12.2 | 4.0-6.7 | 2.7-4.4 |
| NSL/HL | 0.35-0.95 | 0.22-0.51 | 0.17-0.21 | 0.16 | 0.42-0.43 | 0.17-0.66 | 0 | 0.14-0.38 | 0.12-0.15 | 0.24-0.43 | 0.58-0.75 | 0.07-0.18 | 0.21-0.39 | 0.15-0.21 | 0.11-0.18 |
| DS | 2.4-8.7 | 4.9-11.3 | 1.8-2.2 | 1.9 | 3.5-6.8 | 2.0-14.2 | Absent | 2.0-5.5 | 0.8-3.0 | 2.6-10.5 | 6.0-17.7 | 0.58-1.65 | 2.3-8.3 | 2.4-4.2 | 1.7-2.1 |
| DS/HL | 0.15-0.38 | 0.20-0.52 | 0.08-0.09 | 0.06 | 0.16-0.17 | 0.14-0.45 | 0 | 0.09-0.24 | 0.06-0.15 | 0.14-0.51 | 0.41-0.53 | 0.03-0.07 | 0.11-0.26 | 0.09-0.13 | 0.07-0.09 |
| WNC | 0.6-2.9 | 1.0-2.2 | 1.6-2.1 | 1.6 | 2.3-4.1 | 1.8-4.2 | Absent | 1.3-3.4 | 0.9-1.5 | 2.9-4.8 | 3.0-4.8 | ? | 1.4-2.9 | 1.0-1.5 | 1.4-1.6 |
| DIAS | 3.3-5.4 | 1.2-6.8 | 5.0-7.9 | ? | 2.0-6.7 | 2.7-8.3 | Absent | 4.9-8.4 | 2.2-6.3 | 2.6-4.8 | 2.5-5.3 | Absent | 3.6-7.6 | 3.9-6.1 | 5.1-7.6 |
| DIAS/SVL | 0.03-0.05 | 0.01-0.06 | 0.04-0.07 | ? | 0.05 | 0.03-0.09 | Absent | 0.04-0.08 | 0.02-0.08 | 0.02-0.04 | 0.03-0.04 | Absent | 0.05-0.08 | 0.03-0.07 | 0.05-0.07 |
| DIASN | 8-9 | 1-11 | 11-15 | 7 | 4-7 | 6-17 | Absent | 9-25 | 10-14 | 4-8 | 7-10 | Absent | 12-17 | 6-10 | 10-13 |
| FOREL | 36.8-54.2 | 33.7-56.0 | 33.9-61.5 | ? | 54.2-83.8 | 31.7-56.8 | 30.2-35.3 | 35.6-49.8 | 28.2-42.8 | 49.8-56.6 | 58.4-85.0 | ? | 22.3-42.9 | 34.7-43.2 | 38.0-51.7 |
| HINDL | 46.2-72.9 | 39.0- 69.6 | 43.3- 68.6 | ? | 78.5-107.2 | 42.0- 77.1 | 38.4- 47.8 | 48.8- 65.0 | 48.5- 50.4 | 60.4-68.4 | 72.1-129.7 | ? | 38.2- 60.6 | 54.1-63.9 | 48.5- 65.6 |
| SUPRAL | 10-13 | 10-14 | 12 | 12-13 | 10 | 11-15 | 12-13 | 10-13 | 10-13 | 12-14 | 10-11 | 9-12 | 10-12 | 11-14 | 12-13 |
| INFRAL | 9-11 | 12-15 | 11-12 | 11 | 12-13 | 10-14 | 11-13 | 10-12 | 9-13 | 12-14 | 11-12 | 10-11 | 10-12 | 10-14 | 11-12 |
| VENT | 63-66 | 51-68 | 51-55 | 63 | 55-66 | 50-67 | 53-58 | 55-63 | 52-61 | 55-65 | 64-71 | ? | 57-67 | 52-66 | 47-57 |
| FI | 17-23 | 13-17 | 23 | 18 | 16-17 | 15-20 | 13-14 | 16-18 | 17-19 | 15-18 | 16-21 | 14-17 | 15-17 | 19-21 | 20-21 |
| TO | 25-29 | 19-26 | 26-28 | 24 | 22-24 | 20-26 | 17-19 | 21-26 | 22-23 | 21-23 | 20-27 | 19-23 | 21-24 | 25-28 | 23-27 |
| OS | 3.1-10.0 | 4.0-9.4 | 1.2-2.6 | 1.0 | Absent | 4.1-13.6 | Absent | 2.5-4.9 | 3.2-3.4 | Absent | Absent | ? | 2.6-9.5 | 4.5-7.0 | 1.8-2.3 |
| OS/HL | 0.19-0.44 | 0.16-0.43 | 0.10-0.11 | 0.03 | 0 | 0.24-0.56 | 0 | 0.11-0.50 | 0.14-0.15 | 0 | 0 | ? | 0.13-0.30 | 0.16-0.23 | 0.09-0.10 |
| NSSOS | 5 | 4-6 | 6-7 | ? | Absent | 4-6 | 4-5 | 4-6 | 4-5 | Absent | Absent | ? | 4-5 | 4-5 | 4-5 |

…...*continued on the next page*

**TABLE 3.** (Continued)

|  | *A. aurantiacrista*  **sp. n.** | *A. armata* | *A. bintangensis* | *A. brachypoda* | *A. capra* | *A cardamomensis* | *A. coronata* | *A. crucigera* | *A. lepidogaster* | *A. murphyi* | *A. nataliae* | *A. phongdienensis* | *A. phuketensis* | *A. tongbiguanensis* | *A. titiwangsaensis* |
| --- | --- | --- | --- | --- | --- | --- | --- | --- | --- | --- | --- | --- | --- | --- | --- |
| CS | 10-14 | 11-15 | 14-15 | ? | 12-14 | 11-16 | 12-15 | 12-15 | 10-14 | 12-14 | 12-13 | 9-13 | 10-14 | 10-14 | 14-15 |
| RW | 2.5-3.7 | 1.7-4.5 | 3.6-5.3 | 3.5 | 4.2-4.6 | 1.7-4.7 | 0.8-0.9 | 2.7-4.0 | 2.8-3.0 | 3.3-5.1 | 4.6-6.1 | 2.07-2.65 | 2.3-3.8 | 3.3-4.5 | 3.6-5.2 |
| RH | 0.9-2.1 | 0.9-1.8 | 1.7-2.0 | 2.3 | 1.8-2.3 | 1.1-2.2 | 0.5-0.8 | 1.3-2.0 | 1.4-1.5 | 1.2-2.0 | 1.8-2.9 | 1.00-1.32 | 1.1-1.7 | 1.0-2.0 | 1.4-1.8 |
| RS | 4-6 | 5-8 | 5-9 | 5-9 | 5-9 | 7-9 | 9 | 7-8 | 6-7 | 8-9 | 7 | ? | 5-9 | 6-9 | 5 |
| NS | 5-6 | 6-10 | 8 | 9 | 9 | 7-10 | 7-9 | 7-9 | 7-8 | 7-8 | 5-8 | ? | 7-8 | 8-9 | 8 |
| NCS | 11-13 | 10-17 | 10-11 | ? | 9 | 9-17 | 8-11 | 9-12 | 7-11 | 13-16 | 10-14 | ? | 12-13 | 10-13 | 11-12 |
| NSCSL | 6-10 | 6-14 | 7-8 | ? | 7-8 | 7-12 | 5-6 | 7-11 | 7-12 | 7-10 | 8-11 | ? | 8-10 | 7-9 | 9-11 |
| NR | 1-2 | 1-2 | 1 | ? | 1-2 | 1-2 | 3-4 | 1-2 | 1-2 | 3-4 | 1 | ? | 1-2 | 2 | 1-2 |
| NSSLC | 9-13 | 10-22 | 9-12 | ? | 9-11 | 10-19 | 6-11 | 10-14 | 10-18 | ? | 13-16 | ? | 11-14 | 9-13 | 11-14 |
| MW | 1.1-2.5 | 0.8-2.0 | 1.3-1.8 | 2.9 | 1.9-2.2 | 0.2-2.1 | 0.6-1.5 | 1.0-1.5 | 1.2-1.3 | 1.7-2.2 | 2.3-2.9 | 0.87-1.52 | 0.5-1.4 | 1.4-1.9 | 1.4-2.0 |
| MH | 0.8-1.6 | 0.8-2.3 | 1.4-2.1 | 2.1 | 1.7-2.2 | 0.9-2.0 | 1.3-1.6 | 1.1-1.7 | 1.2-1.3 | 1.4-2.0 | 2.0-2.9 | 1.04-1.60 | 0.6-1.6 | 1.2-2.0 | 1.4-2.4 |
| PM | 4 | 3-6 | 4-5 | 4 | 4 | 4-5 | 4-5 | 4 | 5 | ? | 4-5 | ? | 4 | 4-5 | 5 |
| YAS | 1 | 0-1 | 1 | 1 | 1 | 0-1 | 0-1 | 1 | 1 | 0-1 | 1 | ? | 0-1 | 1 | 1 |
| ND | 1 | 0-1 | 1 | 1 | 1 | 1 | 0 | 1 | 1 | ? | 0 | 1 | 1 | 1 | 1 |
| LKP | 1 | 1 | 0 | 1 | 1 | 1 | 1 | 1 | 1 | ? | 0 | 1 | 1 | 1 | 0 |
| BEP | 1 | 0 | 1 | 1 | 1 | 1 | 0 | 1 | 0-1 | 0-1 | 0-1 | ? | 1 | 1 | 1 |
| ESBO | 0 | 0 | 1 | 0 | 0 | 0 | 0 | 0 | 0 | ? | 0 | ? | 0 | 0 | 0 |
| GP | 1-4 | 1 | 3-4 | 0 | 3-4 | 1-4 | 0 | 1-2 | 0-1 | 4 | 3-4 | ? | 0-2 | 1-2 | 2-4 |
| OF | 1 | 1 | 1 | 1 | 1 | 1 | 1 | 1 | 1 | ? | 1 | 1 | 1 | 1 | 1 |
